# A Rare Clinical Presentation of Congenital Superficial Angiomyxoma: A Case Report

**DOI:** 10.1002/ccr3.71164

**Published:** 2025-10-12

**Authors:** Saman Al‐Zahawi, Sara Masoomi, Maryam Ghiasi, Vahidesadat Azhari, Kambiz Kamyab, Faezeh Khorasanizadeh, Ifa Etesami

**Affiliations:** ^1^ Department of Dermatology, Razi Hospital Tehran University of Medical Sciences (TUMS) Tehran Iran; ^2^ Department of Dermatopathology, Razi Hospital Tehran University of Medical Sciences (TUMS) Tehran Iran; ^3^ Department of Radiology, Razi Hospital Tehran University of Medical Sciences (TUMS) Tehran Iran

**Keywords:** congenital superficial angiomyxoma, pedunculated angiomyxoma, SAM, vulvar neoplasm

## Abstract

Angiomyxoma is a rare atypical mesenchymal proliferation with two distinct forms, deep and superficial. Superficial Angiomyxoma tends to be benign with limited skin involvement. Rarely, Superficial Angiomyxoma appears in the vulvar region in the form of cystic, vascular, or tumoral lesions. The onset of vulvar superficial angiomyxoma is variable and ranges from early childhood to the post‐menopausal period. To the best of our knowledge, this report presents the third documented case of vulvar superficial angiomyxoma with an onset at birth and a subsequent gradual increase in size over 2 years.

## Graphical abstract

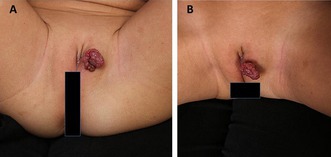

**Summary**

- Pediatric vulvar superficial angiomyxoma is a rare, benign tumor requiring surgical excision and long‐term follow‐up for recurrence and to rule out Carney complex, especially if congenital.

## 1 Introduction

Angiomyxoma is a rare atypical mesenchymal proliferation with two distinct forms, superficial and deep [1]. While superficial angiomyxoma (SAM) is a benign, cutaneous tumor with limited skin involvement, deep angiomyxoma tends to be aggressive with local invasion and rarely distant metastasis [2]. Although initially, SAM was described in association with the Carney complex, sporadic SAM has frequently been recognized. Sporadic SAM is located mainly in the trunk, head, and neck, with a rare occurrence in the vulva. The onset of vulvar superficial angiomyxoma varies and ranges from early childhood to post‐menopausal [3, 4]. The vulvar subtype may have a cystic, vascular, or tumoral appearance. They might be confused with skin tags, labial cysts, and cysts of Bartholin ducts [5]. Importantly, it should be distinguished from deep angiomyxoma when angiomyxoma is vulvar in origin.

Diagnosis depends on clinical suspicion and pathological findings of the diffuse mucinous matrix within the dermis and subcutis, admixed with fibroblasts and collagen fibers in a well‐lobulated lesion [6]. In the vulvar subtype, immunohistochemistry (IHC) for hormonal receptors like estrogen and progesterone may help to exclude deep angiomyxoma [2]. When the IHC is positive for hormonal receptors, it should be followed by an MRI to define the extent of the disease and instruct the surgeon for wide local excision because of a high rate of recurrence [7]. Although the treatment approach for SAM is local excision, similar to deep angiomyxoma, more conservative excision is applied than that for deep angiomyxoma [2]. This report presents a case of vulvar superficial angiomyxoma diagnosed at birth that demonstrated gradual enlargement over 2 years.

## 2 Case History/Examination

A 2‐year‐old female patient presented to our center with a pedunculated mass on the vulva. The vulvar lesion started as a small erythematous nodule at birth. Then, there was a gradual increase in the size of the lesion within 2 years, making the parents apprehensive and prompting them to seek medical consultation. The parents stated that the neonatal and perinatal periods were uneventful, and she had been fully vaccinated with no known medical or surgical events.

**FIGURE 1:**
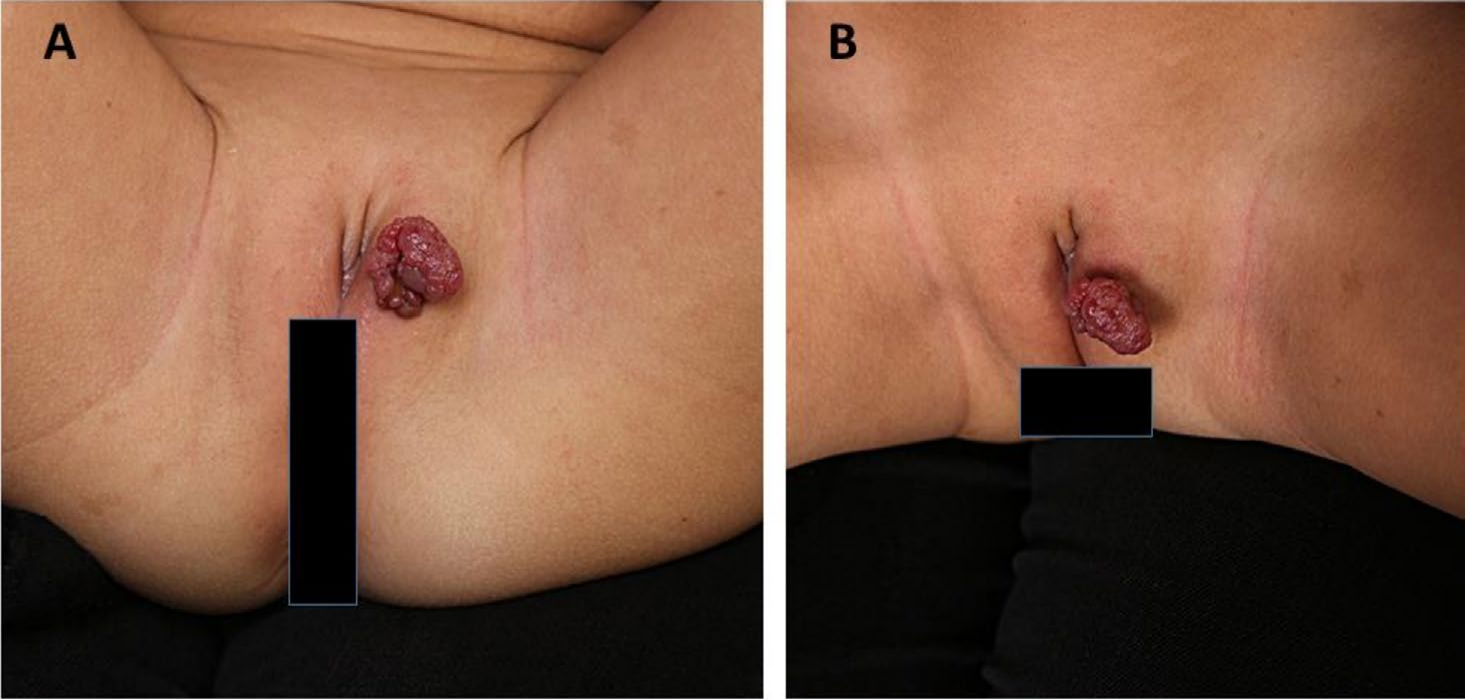
A pedunculated, cauliflower red‐colored, jelly mass in the left vulvar region (A, B).

On examination, there was a 2 × 3 cm, red‐fleshy colored, jelly consistency, cauliflower‐like pedunculated mass on the left vulvar region (Figure 1). On palpation, there was no induration of the mass, and the lesion had no extension to the mucosa of the genitalia. Additionally, there were no widespread nevi or lentiginous lesions on total skin examination.

### 2.1 Differential Diagnosis, Investigations, and Treatment

**FIGURE 2:**
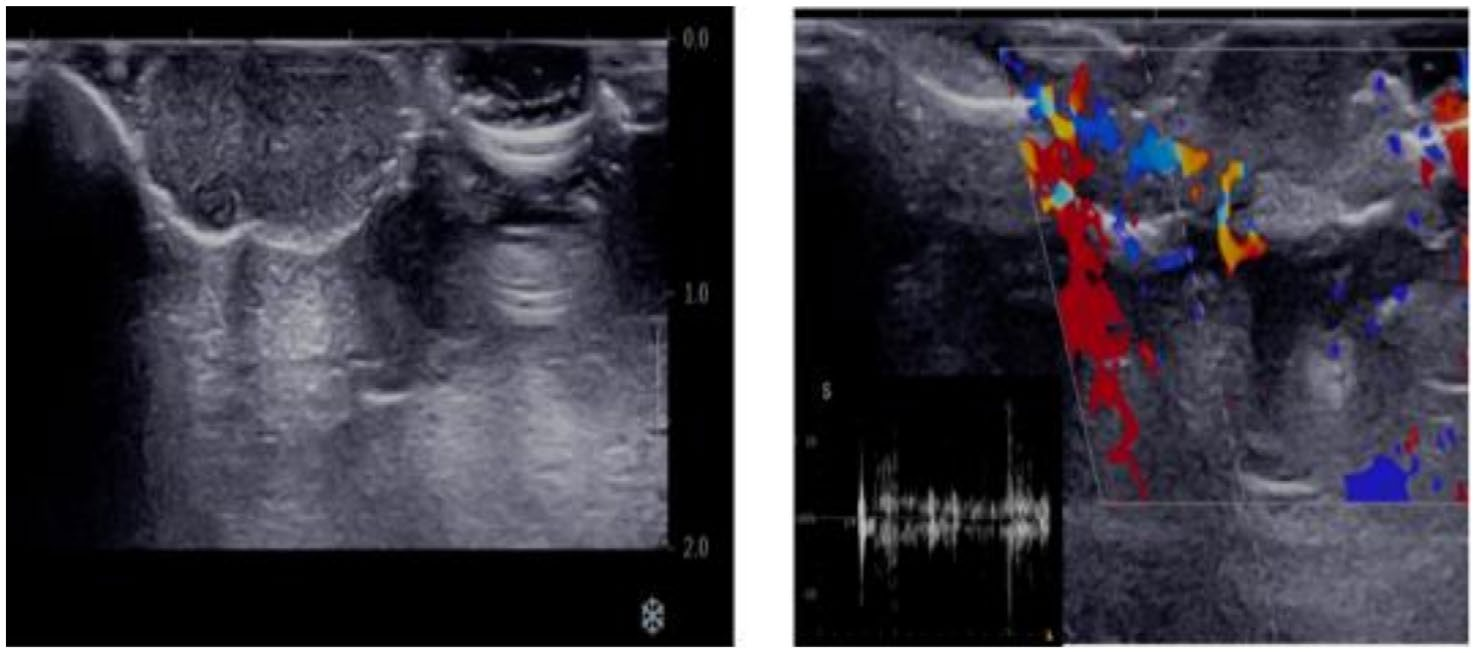
Color Doppler ultrasound using an 18 MHz linear probe revealed a well‐defined hypoechoic pedunculated mass measuring 18 × 18 mm in the vulva, exhibiting mild peripheral and internal vascularity with a peak systolic velocity of approximately 10 cm/s.

Metabolic panel and blood chemistry were normal, and there were no signs of endocrinopathies. Ultrasound was performed to assess the mass before total excision of the lesion for biopsy. Doppler Ultrasound showed an 18 × 18 mm, well‐defined, hypoechoic, pedunculated mass, with mild internal and peripheral vascularity (Figure 2A,B). Total mass excision was performed with a 2 mm free margin and free tension primary closure.

**FIGURE 3:**
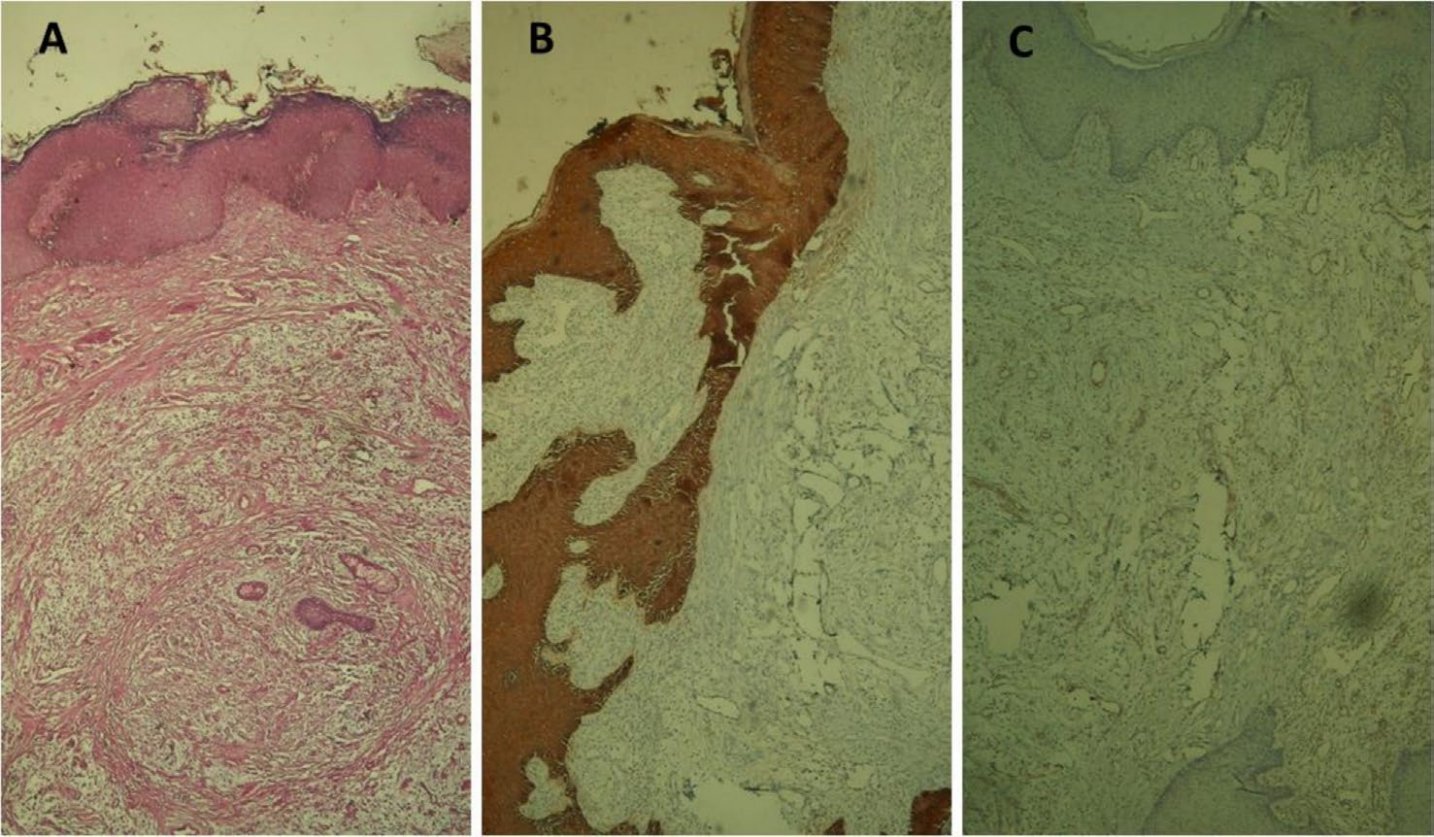
H & E revealed ill‐defined, dermal multinodular neoplastic proliferation composed mainly of satellite‐spindle cells in extensive myxoid stroma, with prominent vasculature (A), IHC showed negative pan‐cytokeratin (B), and staining for desmin was negative (C).

Histopathological evaluation of the specimen revealed a polypoid skin lesion covered by acanthotic epidermis. There was an ill‐defined, dermal multinodular neoplastic proliferation composed mainly of satellite‐spindle cells in extensive myxoid stroma with prominent vasculature. Scattered epithelial strands and keratin‐filled epidermoid cystic spaces were also present. The margin of the excised lesion was free of tumoral proliferation. The clinical‐pathological findings were compatible with superficial angiomyxoma, and the pathologist recommended further evaluation by IHC. IHC was positive for vimentin, CD34, and SMA, but it was negative for desmin, Pan CK, EMA, desmin, and S100 (Figure 3A–C).

### 2.2 Outcome and Follow‐Up

To ensure optimal follow‐up, patients were recommended to return to our center for check‐ups every 3 months during the first year after excision. This monitoring period was designed to detect any recurrent lesions, the presence of multiple nevi, or the appearance of new skin lesions.

## 3 Discussion

Pediatric vulvar superficial angiomyxoma is a benign, sporadic, extremely rare tumor, with only eight cases being reported in the literature (including ours) (Table 1). The vulva is the third most common location of SAM in children after the head and neck [3]. Vulvar lesions of SAM are usually asymptomatic and appear as a painless mass, pedunculated lesion, ulcerated bleeding mass, cerebriform nodule, or vascularized pedunculated mass.

**TABLE 1:** Reported cases of vulvar superficial angiomyxoma in children.

Reported cases	Age at presentation	Onset of the lesion appearance	Symptoms and finding	Size	Treatment
Okada et al.	3‐year	18 months before presentation	Pedunculated polypoid covered by normal skin	Not reported	Excision
Calonje et al.	11‐year	8‐year	Vulval mass	4 cm	Excision
Lee et al.	13‐year	11‐year	Vulval mass with bleeding	7 cm × 3 cm × 2 cm	Excision
Flynn and O'Brien	7‐year	Since birth	Pink‐tan, pedunculated, cauliflower‐like mass	2.5 cm × 1.5 cm × 1.2 cm	Excision
Hafeez et al.	17‐month‐old girl	2–3 months of age	Pink cerebriform nodule	0.5 cm	Excision
Rose and Arredondo	6‐year	Since birth	Exophytic tumor	4.5 × 4.5 × 2.5 cm	Excision
Oral et al.	8‐year	2 weeks after bone marrow transplantation	Painless vulvar mass	1 cm × 1 cm	Excision
Our case	2‐year	Since birth	Exophytic, lobulated, vascularized tumor	2 cm × 3 cm	Excision

The age of onset of pediatric valvar SAM has varied from birth until 11 years, with usual late consultation with physicians until the lesion grows larger or develops ulceration with bleeding. To our knowledge, this is the third case of SAM with an onset at birth and late presentation at 2 years after significant growth of the lesion. The size may be as small as 0.5 cm to as large as 7 cm [8, 9]. Although the literature has defined sporadic SAM as an acquired condition [10], our case is the third case of having lesions at birth, which may point to a sporadic congenital condition unrelated to the known Carney Complex. Nevertheless, children with early‐onset SAM should be evaluated thoroughly for the latter syndrome as angiomyxoma may be the first sign of Carney Complex. Carney Complex is an autosomal dominant condition characterized by skin pigmentation, endocrine overactivity, and myxoma due to a mutation in PRKAR1A. The absence of blue nevi, endocrinopathies, and multiple cutaneous myxomas in different anatomical locations led to the exclusion of the Carney Complex in our study. Other conditions involving the female genitalia in a child should be considered, like rhabdomyosarcoma, liposarcoma, epidermal cyst, abscess, and the aggressive, deep form of vulvar angiomyxoma [11].

Pathological assessment is essential for the definitive diagnosis of clinically suspected cases of pediatric vulvar SAM and differentiation from the aforementioned conditions. The characteristic pathological finding is a well‐defined tumor with diffuse vascular proliferation in an abundant mucinous matrix. IHC is usually recommended to distinguish between the aggressive, deep angiomyxoma, which is hormone‐dependent and is positive for both estrogen and progesterone receptors, from the superficial angiomyxoma, which is CD34+, Vimentin negative, and S100+ [12]. Our case was S100 negative, Vimentin+, and CD34+, findings compatible with superficial angiomyxoma. However, entities with mucin infiltration, like focal cutaneous mucinosis, cutaneous myxoid cyst, dermal nerve sheath myxoma, and myxoid liposarcoma, should be considered in the histopathological differential diagnosis of SAM.

To distinguish the deep angiomyxoma from the vulvar superficial angiomyxoma, radiological evaluation with either MRI or Ultrasound is needed to evaluate the depth of the lesions and their vascular characterization. The aforementioned preoperative imaging aids in the complete surgical resection of the lesion, which is the standard treatment modality. It is noteworthy that incomplete surgical excision accounts for 30%–40% of recurrent cases of angiomyxoma [13]. Also, recurrence in pediatric vulvar SAM after surgical resection has been reported in one patient by Raquel et al. in their systematic review of pediatric vulvar SAM [3]. We recommend longitudinal evaluation of children with pediatric vulvar SAM for recurrence and signs of Carney Complex.

## 4 Conclusion

Pediatric vulvar angiomyxoma is a rare benign condition in children with a variable onset from birth to late adolescence. Lesions are treated by surgical excision. Patients are longitudinally evaluated for recurrence and to exclude the Carney complex.

## Author Contributions

**Saman Al‐Zahawi:** writing – original draft, writing – review and editing. **Sara Masoomi:** writing – original draft, writing – review and editing. **Maryam Ghiasi:** conceptualization, data curation. **Vahidesadat Azhari:** visualization. **Kambiz Kamyab:** visualization. **Faezeh Khorasanizadeh:** visualization. **Ifa Etesami:** data curation, supervision.

## Consent

Written informed consent was obtained from the patient to publish this report following the journal's patient consent policy.

## Conflicts of Interest

The authors declare no conflicts of interest.

## Data Availability

The authors elect to share data upon request.
